# Cationic High Molecular Weight Lignin Polymer: A Flocculant for the Removal of Anionic Azo-Dyes from Simulated Wastewater

**DOI:** 10.3390/molecules23082005

**Published:** 2018-08-11

**Authors:** Shoujuan Wang, Fangong Kong, Pedram Fatehi, Qingxi Hou

**Affiliations:** 1 Key Laboratory of Paper Science and Technology of Ministry of Education, Qilu University of Technology (Shandong Academy of Sciences), Jinan 250353, China; nancy5921@163.com; 2 Department of Chemical Engineering, Lakehead University, 955 Oliver Road, Thunder Bay, ON P7B 5E1, Canada; 3 Tianjin Key Laboratory of Pulp & Paper, Tianjin University of Science & Technology, Tianjin 300222, China; qingxihou@tust.edu.cn

**Keywords:** lignin-METAC, lignin modification, azo dye, flocculation, COD

## Abstract

The presence of dyes in wastewater effluents made from the textile industry is a major environmental problem due to their complex structure and poor biodegradability. In this study, a cationic lignin polymer was synthesized via the free radical polymerization of lignin with [2-(methacryloyloxy) ethyl] trimethyl ammonium chloride (METAC) and used to remove anionic azo-dyes (reactive black 5, RB5, and reactive orange 16, RO16) from simulated wastewater. The effects of pH, salt, and concentration of dyes, as well as the charge density and molecular weight of lignin-METAC polymer on dye removal were examined. Results demonstrated that lignin-METAC was an effective flocculant for the removal of dye via charge neutralization and bridging mechanisms. The dye removal efficiency of lignin-METAC polymer was independent of pH. The dosage of the lignin polymer required for reaching the maximum removal had a linear relationship with the dye concentration. The presence of inorganic salts including NaCl, NaNO_3_, and Na_2_SO_4_ had a marginal effect on the dye removal. Under the optimized conditions, greater than 98% of RB5 and 94% of RO16 were removed at lignin-METAC concentrations of 120 mg/L and 105 mg/L in the dye solutions, respectively.

## 1. Introduction

Dyes are readily found in wastewater effluents of various industries including: dye manufacturing, textile, cosmetic, pharmaceuticals, food, rubber, leather, printing, and pulp & paper. Dyes are classified into acidic, basic, azo, diazo, disperse, metal complex, and anthraquinone-based categories [1,2]. There are approximately 8000 dyes and 10,000 commercial dye-based products in the market [3,4]. Azo-dyes are aromatic compounds with one or more azo bonds (–N=N–) and currently represent 60–70% of commercially used dyes in the world [5,6]. They are generally used for coloring plant fibers including cotton, hemp and linen, wool fibers, as well as inorganic particles (e.g., clay) [5,6]. In the coloring process, not all of the dye is adsorbed onto the end-use products, and as a result, some remain in the process effluents (i.e., wastewater), which must then be treated [7]. In addition, dyes may cause serious health problems, such as allergy, dermatitis, skin irritation, and cancer [8]. The identification of an effective chemical treatment for the removal of dyes from wastewater is currently needed. 

Presently, many chemical and physical treatment methods are used for treating effluents, including chemical oxidation (using H_2_O_2_, ozone), electrolysis, biodegradation (aerobic and anaerobic), adsorption (activated carbon or biosorbents) [9,10,11], coagulation, flocculation, and their combinations [5,7,12,13,14]. However, the removal of dyes is a challenging task and many small organic molecules remain in the effluent after partial decomposition using the above degradation treatment processes. Coagulation/flocculation can be used as an effective method for dye removals, as it can precipitate the soluble dye molecules (through charge neutralization and/or bridging), is cost-effective, energy efficient, and easy to use [14,15,16]. When separated from the solution in floc forms, the isolated flocs can be oxidized chemically or biochemically to decompose. In other words, the interaction of a flocculant and dye in solution facilitate the separation of dyes, which can subsequently be decomposed by oxidation, for instance. 

Commercial coagulants and flocculants include inorganic salts as well as synthetic and natural organic polymers. Inorganic coagulants consisting of aluminum sulfate, ferric chloride, and polyaluminum chloride usually require a large dosage and produce a high amount of sludge [17,18]. Synthetic flocculants, e.g., polyacrylamides, are extensively used because of their superior performance and limited sludge production [19]. However, the high price of the synthetic polymers and their limited biodegradability are their main drawbacks [19]. Natural polymers, such as chitosan and polysaccharides, are also used as flocculants because of their low price and biodegradability, but they are not shear stable, have limited removal efficiency, and require a high dosage to reach an acceptable flocculation efficiency [20]. To overcome the shortcomings of both synthetic and natural flocculants, the syntheses of semi-natural polymers have been investigated [21,22,23,24]. In one study, a carboxymethyl chitosan-graft-polyacrylamide copolymer with a 74% grafting ratio was able to remove 92% of dyes from an aqueous solution [21]. In another study, the grafting of (2-methaceyloyloxyethyl) trimethyl ammonium chloride on chitosan was produced, and the product was used as a flocculant for wastewater treatment (95.2% turbidity removal) [22]. Xylan-METAC copolymer with grafting ratio of 198% has also been prepared and used to remove dyes from wastewater (97.8% dye removal efficiency) [25].

Lignin is the second most abundant natural material and is currently an under-valued co-product of pulping and biofuel industries. Lignin has great potential to be converted into a flocculant for the dye removal [26,27]. Alternatively, a cationic lignin with a small molecular weight was prepared by grafting glycidyl-trimethylammonium chloride onto lignin and was used to remove anionic dyes from wastewater with a 95% dye removal efficiency [26]. In the present study, lignin and [2-(methacryloyloxy) ethyl] trimethylammonium chloride (METAC) were polymerized in an acidic aqueous solution through a heterogeneous reaction to produce a high molecular weight cationic lignin [27]. The first objective of this study was to investigate the impact of lignin-METAC polymer as a flocculant for dye removals. 

It was comprehensively discussed in the literature that the properties of polymers significantly affect their interaction with colloidal particles in solutions/suspensions [28]. In this regard, it is unclear how the properties of lignin-METAC polymer would affect its flocculation performance. The second objective of this study was to investigate the impact of lignin-METAC polymer’s properties on its flocculation efficiency. The main novelty of this work was the application of lignin-METAC polymer as a flocculant in simulated dye solutions. As stated earlier, the removal of dyes was affected by the pH, salt, and dye concentration in solutions [27,29,30]. In this work, the effects of pH, salt, dye concentration, charge density and molecular weight of lignin-METAC polymer on the dye removal efficiency were fundamentally examined. 

## 2. Results and Discussion

### 2.1. Cationic Lignin Preparation

Lignin-METAC was prepared in an aqueous heterogeneous reaction by the free radical polymerization of kraft lignin and METAC under mild acidic condition initiated by K_2_S_2_O_8_. During this reaction, METAC monomers, which contain cationic quaternary ammonium groups, were grafted onto the lignin backbone, yielding a cationic lignin with a high molecular weight. As shown in Table 1, increasing the METAC/lignin molar ratio in the polymerization reaction increased both the charge density and the molecular weight of lignin-METAC. It has been reported in the literatures that dye removal through flocculation occurs via charge neutralization, bridging, and hydrophobic/hydrophobic interaction [17,31,32,33]. Therefore, an increase in both the charge density and molecular weight of lignin after polymerization will affect its flocculation performance for dye molecules. 

### 2.2. Effects of Dosage and pH

**Figure 1:**
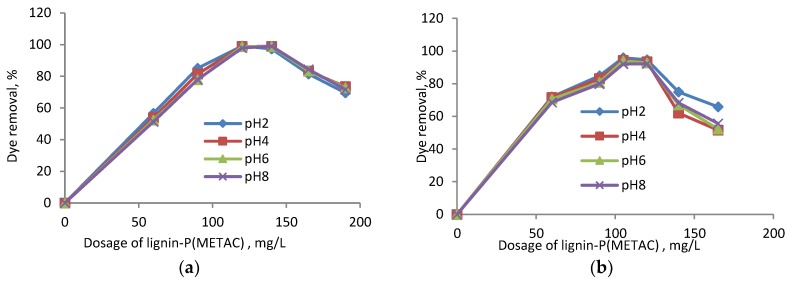
Effect of pH on the dye removal of (**a**) RB5; (**b**) RO16 (from a dye concentration of 100 mg/L) using Sample 4.

The interaction between dye and polymer segments can be affected by the pH of the effluent wastewater [23]. The impacts of the dosage of lignin-METAC (sample 4) and the pH of the solution on the dye removal are shown in Figure 1. Regardless of the pH, the dye removal efficiency reached a maximum at 120 mg/L and 105 mg/L of lignin-METAC concentration for RB5 and RO16, respectively. In this case, the sulfonate groups (i.e., anionic groups) of the dye were neutralized by the cationic ammonium groups of lignin-METAC polymer forming large flocs that settled [34]. When the concentration of lignin-METAC polymer was higher than 150 mg/L for RB5 and 120 mg/L for RO16, more lignin-METAC interacted with the dye segments generating coagulates that were probably positively overcharged by lignin-METAC. These coagulates could repel each other in the solutions as they had a net charge. The balance of the repulsion of the coagulates in the solutions would yield the stabilization of the coagulates (and thus, dye segments) in the solutions, decreasing the dye removal efficiency [24].

The limited impact of pH on dye removal is due to the fact that, although the positive charge of lignin-METAC would be reduced with increasing pH as the lignin-METAC are surrounded with OH^-^ counter ions, the cationic charge density of the lignin-METAC polymer would still be sufficiently high to act as an effective flocculant under alkaline conditions. 

Moreover, the high molecular weight of lignin-METAC can also facilitate the removal of dye through a bridging effect. A similar trend was reported using carboxymethyl cellulose-graft-poly[(2-methacryloyloxyethyl) trimethyl ammonium chloride] (CMC-g-METAC) as a flocculant to remove acid green dye, and pH had a minimal effect in the dye removal [24]. It can be claimed that the dye removal efficiency of lignin-METAC polymer with a high charge density and a high molecular weight is independent of pH. 

**Figure 2:**
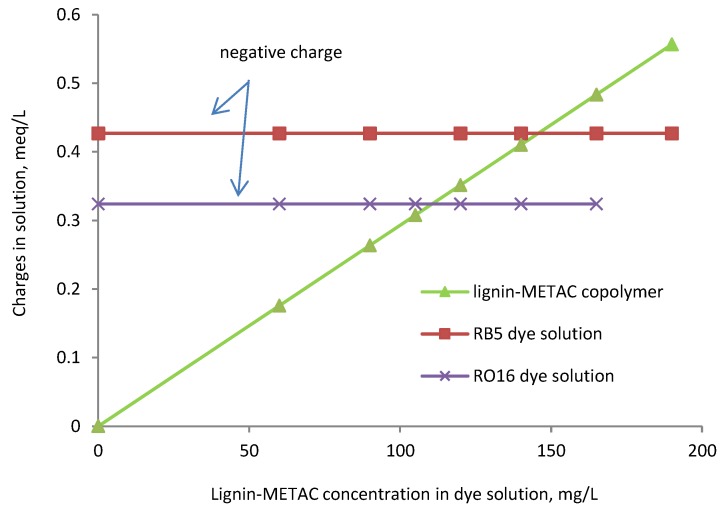
Overall cationic charges of the dye solutions (dye concentration, 100 mg/L) as function of lignin-METAC polymer (sample 4) concentration in the solutions.

The total cationic charge (meq/L) of lignin-METAC polymer in the dye solution was determined and is shown in Figure 2. The total cationic charges introduced to the solution were increased by the addition of the lignin polymer into the solution. Charge neutralization, i.e., point of zero charge, will occur when the total number of charges introduced by the polymer will be equal to the total charges of the dye in the solutions (i.e., the crossing points of lignin-METAC line and dye line shown in Figure 2). This point corresponds to the lignin-METAC concentration of 145.7 mg/L for RB5 and 110 mg/L for RO16, respectively. The optimal experimental concentration of lignin-METAC was 120 mg/L for RB5 and 105 mg/L for RO16 (Figure 1), respectively, which is lower than that of the theoretical concentrations (Figure 2). This provides evidence that, in addition to charge neutralization, other factors, such as bridging, contributed to the interaction of lignin-METAC with the dye molecules in the solutions [25,26,27].

**Figure 3:**
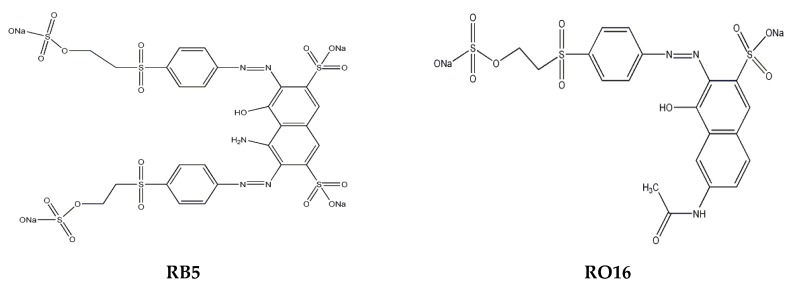
Structure of RB5 and RO16.

It is also apparent that a lower dosage of lignin-METAC polymer was needed to remove RO16 than RB5. This higher efficiency is most likely attributed to the different molecular structures and charge densities of RB5 and RO16. As shown in Figure 3, RO16 has two sulfonate groups, while RB5 contains four sulfonate groups, requiring a higher concentration of lignin-METAC polymer to neutralize its charge. A similar phenomenon has also been observed by Szygula and coworkers in removing sulfonated azo-dyes by using chitosan from solutions [23]. 

### 2.3. Effect of Dye Concentration

**Figure 4:**
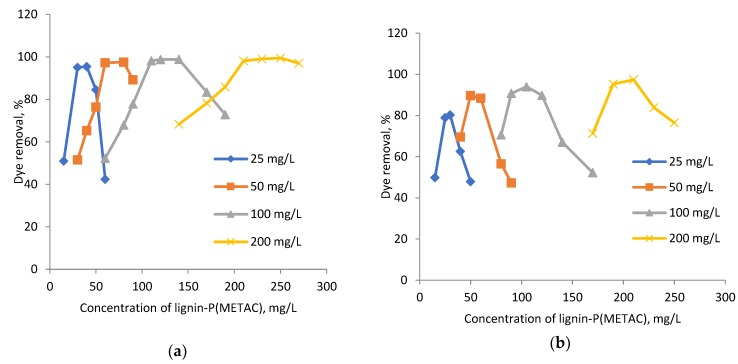
Effect of dye concentration on dye removal (**a**), RB5; (**b**) RO16 at pH 6 using sample 4.

The effect of dye (25, 50, 100, 200 mg/L) and lignin-METAC concentration on the removal of RB5 and RO16 at pH 6, which is the typical pH of dye effluent, is shown in Figure 4 [35]. The concentration of lignin-METAC polymer was varied in order to determine the best concentration required for the maximum dye removal. The dye removal efficiency increased and then decreased with increasing the concentration of lignin-METAC polymer for both dye solutions and an optimum dosage of 120 mg/L and 105 mg/L for RB5 and RO16, respectively, was determined.

**Figure 5:**
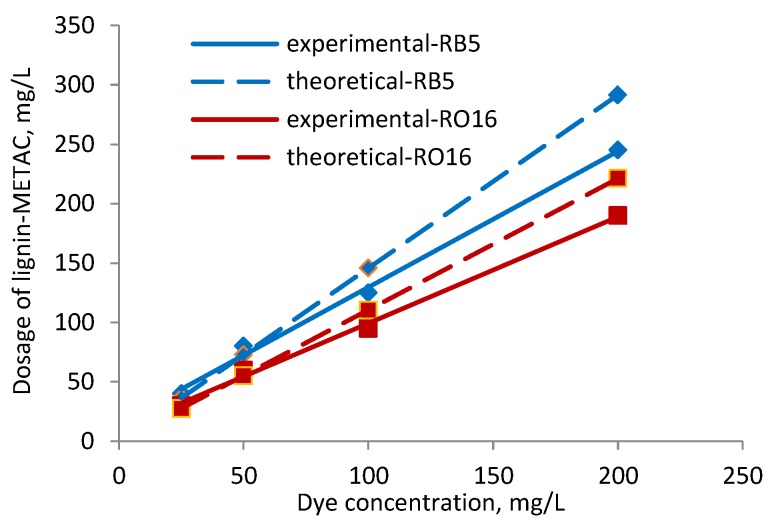
Relationship between optimum lignin-METAC dosage and dye concentration.

From the results shown in Figure 4, the amount of lignin-METAC needed to remove the maximum amount of dye was determined and presented as a function of dye concentration in Figure 5. In this figure, the theoretical amount of lignin-METAC polymer required for dye neutralization is also shown. It is apparent that, at the same dye concentration, less lignin-METAC polymer was needed to remove RO16 than RB5. As listed in Table 2, RO16 had a lower anionic charge density than RB5, which is due to its smaller number of sulfonate groups. Therefore, a lower concentration of lignin-METAC polymer was necessary to neutralize the anionic charges of RO16. The theoretical and experimental correlations in Figure 5 depict that (1) at a lower dye concentration, charge neutralization was the main cause for dye removal, as the theoretical and experimental values were very close; and (2) at a high dye concentration, less lignin-METAC was necessary experimentally (than theoretically) to remove the dye, illustrating that bridging played a significant role in dye removal at higher concentrations.

The correlation between the dye removal and lignin-METAC concentration is listed in Table 3. A linear correlation with a high regression was obtained in both cases. A stoichiometric correlation was obtained between lignin-METAC and dye, indicating a close to one to one charge interaction.

### 2.4. Effect of Charge Density and M_w_ of Lignin-METAC Polymer

**Figure 6:**
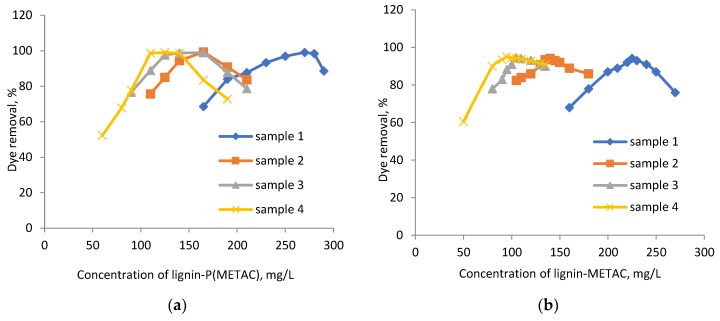
Effect of dosage of lignin-METAC samples with different charge densities on dye removal (100 mg/L RB5 (**a**) and RO16 (**b**) dye solution, pH 6, 30 °C).

The effect of charge density and M_w_ of lignin-METAC on dye removal was presented in Figure 6. A 100 mg/L sample of RB5 and RO16 solutions were used as the simulated dye wastewater in this experiment. At the maximum dye removal, a smaller dosage of lignin-METAC polymer with a higher charge density and higher M_w_ was needed for both RB5 and RO16 solutions. 

**Figure 7:**
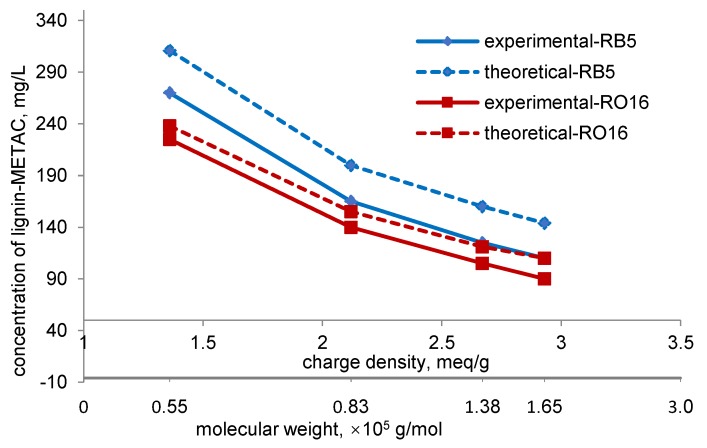
Relationship between charge density, MW, and optimum concentration of lignin-METAC copolymer in dye removal.

Based on the charge densities of the dyes in Table 2 and that of the lignin-METAC in Table 1, the concentration of lignin-METAC polymer that could result in a maximum dye removal was determined and is shown as a function of dye removal in Figure 7. The concentration of lignin-METAC to obtain the maximum dye removal was 270 mg/L, 165 mg/L, 125 mg/L, and 110 mg/L for RB5 and 225 mg/L, 140 mg/L, 105 mg/L, and 90 mg/L for RO16 for samples 1 to 4, respectively. Interestingly, the concentration of lignin-METAC used experimentally was lower than that required theoretically to interact with the dyes, which was due to the bridging effect of dyes as the bridging effect is discounted in the theoretical estimation. Moreover, the higher the charge density and molecular weight of lignin-METAC, the larger difference there was between the experimental and theoretical concentrations, further illustrating that the bridging effect was more pronounced when the polymer had a higher molecular weight. The bridging effect in dye removal from wastewater has also been reported in the literature when removing acid violet 5, methyl orange, and acid black 1 from dye wastewater [21,27,36].

### 2.5. Effect of Inorganic Salts

**Figure 8:**
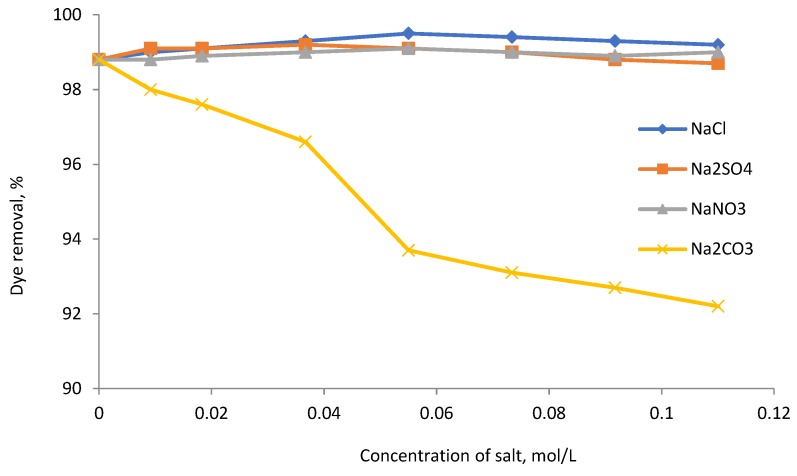
Effect of salt dosage on dye removal (100 mg/L RB5 dye solution, 110 mg/L lignin-METAC dosage (sample 4), pH 6, 30 °C).

Common inorganic salts, such as chloride, sulphate, carbonate, and nitrate, present in textile effluents may affect dye removal [23,29]. For this reason, the effect of NaCl, Na_2_SO_4_, Na_2_CO_3_, and NaNO_3_ on the dye removal of RB5 (100 mg/L), which has a higher dye removal percentage than RO16, was investigated in Figure 8. The addition of NaCl, Na_2_SO_4_, and NaNO_3_ to the solution did not have a significant effect on the removal of RB5 dye. The percentage of dye removal remained constant at high salt concentrations, illustrating the high efficiency of lignin-METAC as a flocculant. However, the removal of RB5 was more influenced by Na_2_CO_3_. In this case, the dye removal efficiency decreased from 98.8% to 92.2%. This has also been reported in the removal of azo-dyes using chitosan and is attributed to a charge screening effect and/or change of the double layer surrounding the flocculated molecules [24]. 

**Figure 9:**
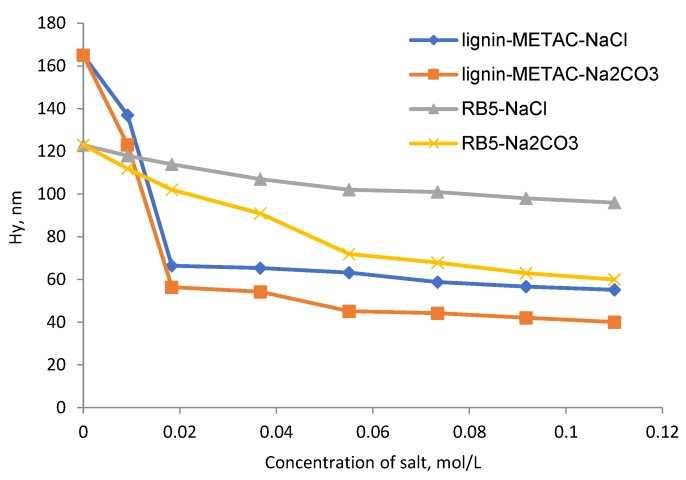
Hydrodynamic diameter (Hy) of lignin-METAC (sample 4) and dye RB5 in solutions containing NaCl or Na_2_CO_3_, pH 6, 30 °C.

In Figure 9, the effect of Na_2_CO_3_ and NaCl on the hydrodynamic diameter (Hy) of lignin-METAC and RB5 are shown. The hydrodynamic diameter of lignin-METAC decreased with an increase in Na_2_CO_3_ and NaCl concentrations, demonstrating that the charge of the lignin-METAC and dye segments were partially screened with increasing ionic strength. The reduced Hy implies that the polymer and dye had coiled structures at high salt concentration [11,37]. When Na_2_CO_3_ was added to the lignin-METAC and dye solutions, a smaller hydrodynamic diameter was observed compared to the solution containing NaCl of both the polymer and dye. This indicates a stronger screening effect of CO_3_^2−^ than Cl^−^, which is consistent with the results presented in Figure 8. The decrease in the dye removal induced by Na_2_CO_3_ is ascribed to two facts: (i) the charges of lignin-METAC and dye are partially screened, resulting in weakened electrostatic interactions between the polymer and the dye, and (ii) a coiled molecule conformation (smaller Hy). These factors would affect the neutralization and bridging of the polymer. This behavior was also reported on the application of a cellulose-based flocculant for removing anionic dye acid green 25 [29].

### 2.6. COD Removal

**Figure 10:**
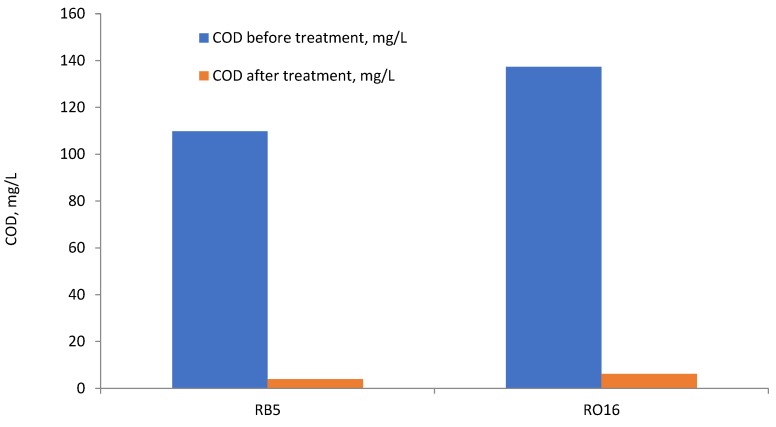
The COD removal of 100 mg/L RB5 and RO16 by 120 mg/L lignin-METAC dosage (sample 4) for RB5 and 105 mg/L lignin-METAC (sample 4) dosages for RO16 pH 6, 30 °C.

The chemical oxygen demand (COD) is an indicator of the load of organics present in wastewater effluents, which is closely monitored for determining the quality of wastewater worldwide [3]. The impact of lignin-METAC on the COD removal from the dye solutions are presented in Figure 10. The concentration of lignin-METAC was based on the optimum dosage obtained in Figure 1. A dosage of 120 mg/L of lignin-METAC in a 100 mg/L RB5 dye solution led to 96.4% COD removal. Alternatively, a dosage of 105 mg/L of lignin-METAC in a 100 mg/L RO16 solution led to 95.5% COD removal. The significant decrease in COD is due to the removal of dye from the solution. Figure 10 also illustrates that little remained of the flocculant, lignin-METAC, in the treated dye solutions as the COD levels of the treated samples were negligible.

## 3. Materials and Methods

### 3.1. Materials 

Softwood kraft lignin was produced by LignoForce^TM^ technology of FPInnovations in its pilot plant facility located in Thunder Bay, ON, Canada [38]. [2-(Methacryloyloxy) ethyl] trimethylammonium chloride solution (METAC), 80 wt.% in H_2_O, potassium persulfate (K_2_S_2_O_8_, ACS reagent ≥ 99.0%), NaCl, NaNO_3_, Na_2_SO_4_, Na_2_CO_3_, and the dyes were all purchased as reagent grade from Sigma-Aldrich (Darmstadt, Germany) company and used as received. The details of reactive black 5 (RB5) and reactive orange 16 (RO16) dyes are presented in Table 2. Anionic polyvinyl sulfate (PVSK) with an M_w_ of 100,000–200,000 g/mol (97.7% esterified) was purchased from Wako Pure Chem. Ltd., Osaka, Japan. Ethanol (95 vol.%) was received from Fisher Scientific (Waltham, MA, USA).

### 3.2. Preparation of Cationic Lignin-METAC

The preparation of lignin-METAC polymer was carried out according to our previously described methods [27]. We comprehensively discussed that METAC would graft to the phenolic OH of lignin and then proceed with chain extension in a free radical polymerization system. We also illustrated that lignin-METAC and xylan-METAC were more effective than polyMETAC as flocculants for clay suspensions [25,27]. In this set of experiments, 1 g of lignin was mixed with 30 mL of deionized water in a 250 mL three-neck glass flask at 80 °C in a water bath. The suspension was purged with nitrogen gas for 30 min, and then a determined amount of METAC was added to the suspension based on the molar ratio of METAC to lignin (the molecular weight unit of lignin was assumed 180 g/mol) [27]. Then, the pH was adjusted to 4. A 5 mL solution of K_2_S_2_O_8_ (0.03 g) was then added dropwise to the reaction mixture to initiate the polymerization. The reaction was heated to 80 °C for 3 h and then cooled to room temperature. Subsequently, the reaction mixture was poured dropwise into a 95 vol.% ethanol solution in order to precipitate the lignin-METAC polymer from the rest of the reaction medium [27]. The suspension was then centrifuged at 2100× *g* for 10 min using a Sorvall ST 16 laboratory centrifuge in order to separate the lignin-METAC polymer from the suspension. The lignin-METAC polymer was then dried in a 105 °C oven prior to use. However, drying may affect the properties of lignin-METAC, and thus drying of this polymer using other methods, e.g., freeze drying or vacuum drying, is suggested. The properties of lignin-METAC polymers with varying amount of METAC along with the reaction conditions are listed in Table 1.

### 3.3. Charge Density Analysis

Approximately 0.05 g of lignin-METAC polymer and dye were separately dissolved in 50 g of water, the solutions were then immersed in a water bath shaker (Innova 3100, Brunswick Scientific, Edison, NJ, USA) and shaken at 150 rpm and 30 °C for 2 h. The charge densities of the samples were then measured using a Particle Charge Detector, Mütek PCD 04, with a 0.005 M PVSK solution. The charge densities of the dyes can be found in Table 2, while that of lignin-METAC polymer are shown in Table 1.

### 3.4. Preparation of Dye Solutions

The dye solutions were prepared by dissolving a specific amount of dye in deionized distilled water to make up the dye solutions with varying concentrations (25, 50, 100, 200 mg/L) at different pH (2, 4, 6, 8). The solutions were kept overnight stirring at 200 rpm and room temperature. The dye solutions were considered as the simulated wastewater effluents in this work. 

### 3.5. Hydrodynamic Diameter (Hy) Measurement

The hydrodynamic diameter (Hy) of the RB5, RO16 and lignin-METAC polymer was determined using a dynamic light scattering analyzer (DLSA), BI-200SM Brookhaven Instrument, NY, USA at a scattering angle of 90°. The light source for the DLSA is a power solid state laser with a maximum power of 35 mW and a wavelength of 637 nm. To measure the hydrodynamic diameter, a 0.02 g/L sample of lignin-METAC polymer and 100 mg/L of dye solution were stirred for 30 min and then a specific amount of salt was added into the solutions. The salt-containing solutions were kept at room temperature for 24 h. Subsequently, the solutions were filtered with 0.45 μm Acrodisc syringe filters and then tested with the instrument.

### 3.6. Dye Removal Analysis

In this set of experiments, 1 g/L aqueous solution of lignin-METAC polymer was prepared with deionized distilled water at room temperature. Different amounts of lignin-METAC were then added to 30 mL of dye in centrifuge tubes as seen in Figure 4 and Figure 6. The tubes were then immersed in a water bath shaker at 30 °C and 150 rpm for 10 min. The tubes were then centrifuged at 1500× *g* for 10 min using a Sorvall ST 16 centrifuge. The filtrates were collected and the concentration of dye remaining in the filtrates was measured using calibration formulas by a UV/Vis spectrophotometer (Genesys 10s). The dye removal was calculated based on equation 1 [20,39,40]:

(1) $$\;\mathrm{Dye}\;\mathrm{removal}=\;\frac{A_{0}-A}{A_{0}}\;\times 100\;$$

where A_0_ and A are the absorbance of the dye solutions (Table 2) before and after the addition of lignin-METAC. The chemical oxygen demand (COD) of the simulated dye solutions (100 mg/L of dye concentration) was measured before and after the addition of lignin-METAC using YSI CR2200 COD thermo reactor. The COD determination is based on the amount of potassium dichromate reduced in concentrated sulfuric acid after 2 h at 150 °C. The test tubes used were manufactured by the Hach Company (Loveland, CO, USA). The COD removal was determined using equation 2:

(2) $$\;\mathrm{COD}\;\mathrm{removal}=\;\frac{C_{0}-C}{C_{0}}\times 100\;$$

where C_0_ and C are the COD of dye solutions before and after lignin-METAC polymer treatment, respectively.

## 4. Conclusions

The cationic lignin-METAC polymer was an effective flocculant for removing anionic dye from simulated wastewater. The results showed that charge neutralization and bridging effects were the main mechanisms for the dye removal. Increasing the charge density and molecular weight of the lignin-METAC polymer improved the efficiency of lignin-METAC polymer for the dye removal. The presence of inorganic salts including NaCl, NaNO_3_, and Na_2_SO_4_ in the dye solution did not affect the dye removal efficiency of lignin-METAC, whereas Na_2_CO_3_ did have a slight affect and decreased the dye removal efficiency from 98.8% to 92.2%. The pH had a minimal impact on dye removal and the lignin-METAC polymer was more effective in removing RO16 than RB5. The relationship between the optimum dosage of lignin-METAC and dye concentration was linear and there was a stoichiometric interaction between the dye and lignin-METAC. Furthermore, more than 95% of COD was removed by treating the dye solutions with lignin-METAC polymer at the optimized dosages.

## Figures and Tables

**Table 1:** Reaction conditions and physical properties of Lignin-METAC polymer.

Lignin-METAC Copolymer	METAC/Lignin Molar Ratio	Charge Density, meq/g	M_n_, ×10^6^ g/mol	M_w_, ×10^6^ g/mol	M_w_/M_n_
Sample 1	0.8	1.36	0.32	0.55	1.718
Sample 2	1.0	2.12	0.45	0.83	1.844
Sample 3	1.3	2.67	0.96	1.38	1.438
Sample 4	1.6	2.93	1.15	1.65	1.434

**Table 2:** Physical properties of dyes.

Dye	Molecular Formula	Mw, g/mol	Purity, %	λmax, nm	Anionic Charge Density, meq/g
RB5	C_26_H_21_N_5_Na_4_O_19_S_6_	991.82	55	597	4.27
RO16	C_20_H_17_N_3_Na_2_O_11_S_3_	617.54	≥70	493	3.24

**Table 3:** Correlation between dye removal and lignin-METAC concentration.

Dye	Linear Correlation	R²
RB5	y = 1.1443x+ 15.217	0.9960
RO16	y = 0.9426x+ 4.1304	0.9963

x: dye concentration, mg/L; y: lignin-METAC concentration, mg/L; R^2^: linear correlation coefficient.
